# Biodegradable polydioxanone stents in the treatment of adult patients with tracheal narrowing

**DOI:** 10.1186/s12890-015-0160-6

**Published:** 2015-12-21

**Authors:** Ludek Stehlik, Vladislav Hytych, Jana Letackova, Petr Kubena, Martina Vasakova

**Affiliations:** Department of Respiratory Medicine, Charles University 1st Faculty of Medicine and Thomayer Hospital, Videnska 800, Prague 4, 140 59 Czech Republic; Department of Thoracic Surgery, Thomayer Hospital, Videnska 800, Prague 4, 140 59 Czech Republic; ELLA-CS Ltd., Milady Horakove 504/45, Trebes, 500 06, Hradec Kralove, Czech Republic

**Keywords:** Interventional pneumology, Stents, Tracheal stenosis, Bronchoscopy, Polydioxanone

## Abstract

**Background:**

Biodegradable stents that disintegrate after a period of time are expected to be well tolerated and have advantages over other stents that are more commonly used in practice today. Polydioxanone is a biodegradable polymer that is widely used during surgery with absorbable sutures.

**Methods:**

We present cases from the first four patients to undergo a tracheal polydioxanone stent insertion. Indications included significant non-malignant tracheal stenosis in cases where primary surgical treatment was not possible. The stents were implanted using rigid bronchoscopy and patients received regular follow-ups as needed. This use of biodegradable stents in adult patients was a novel, previously untested approach. The study was approved by the Institutional Ethics Committee and was based on a project entitled; “Biodegradable stents in the management of stenoses of large airways” (project NT 14146-3/2013).

**Results:**

Six biodegradable stents were implanted in four patients with benign stenoses. No technical difficulties occurred and no serious or life-threatening events were recorded. All patients reported some benefit from treatment.

**Conclusion:**

Polydioxanone tracheal stents can be considered when a need for temporary support is expected, and as an alternative to other stents if the latter could compromise the patient. Owing to limited experience and observed disadvantages, further research is needed to fully assess this treatment.

**Trial registration:**

This work is based on project NT14146 - Biodegradable stents in the management of stenoses of the large airways (2013–2015, MZ0/NT), registered from May 1, 2013 in The Research and Development and Innovation Information System of the Czech Republic and in ClinicalTrials.gov, reg. no. NCT02620319, December 2, 2015.

## Background

Stenting is an important therapeutic modality for the maintenance of lumen patency in many tubular organs, including the trachea. Several types of stents are currently available and can be categorized according to the following materials: silicone or other polymer stents, self-expandable metal stents, and hybrid polymer-metal stents (i.e. covered self-expandable metal stents) [1, 2]. Silicone stents are inserted using rigid bronchoscopy; they are well-tolerated, easily removable, and flexible. However, they disturb airway mucociliary clearance and are prone to migration. Other complications associated with silicone stents include granuloma formation that narrows both ends of the stent, and obstructions secondary to trapped secretions. Self-expanding metal airway stents can be inserted using flexible bronchoscopy and, if not covered, affect mucociliary transportation to a lesser degree than is observed with silicone stents. However, with uncovered stents, the stent mesh can become overgrown with tracheal epithelium, which can make removal difficult. These stents also induce a significant mucosal inflammatory response. Additionally, they can fragment, extrude, and penetrate into neighboring structures, such as the esophagus or large vessels. The use of uncovered metal stents in benign stenosis has been limited and controversial [2, 3].

The ideal airway stent has yet to be developed [1, 2, 4, 5]. Biodegradable (BD) stents are made of knitted polymer fibers that degrade when placed in the body; extraction of the device is, therefore, unnecessary. Several in vitro and in vivo studies of tracheal BD stents composed of various materials have been conducted [5–14].

Polydioxanone is a biodegradable polymer in the polyester family, which has attracted a lot of interest due to its exquisite biocompatibility and is currently available on the market in the form of absorbable suture material. It is degraded by hydrolysis (of its ester bonds), which is accelerated under low pH conditions, into harmless degradation products. Polydioxanone tracheal stents appear to be well tolerated by the tracheal mucosa, retain their mechanical strength for as long as 6 weeks, and, in animal models, completely degrade after approximately 15 weeks [10]. They have been successfully used in humans as mechanical support for tracheal transplants, during treatment of obstructive airway complications after lung transplantation, and in children with airway stenosis [15–18]. This work presents our preliminary experience with the use of polydioxanone stents in adult patients suffering from different types of tracheal stenosis. At the time of this study, this particular indication for BD stents was novel and had not been tested previously.

## Methods

We present cases from the first four patients treated with BD polydioxanone stents between September 2013 and December 2014 (including an adequate follow-up period) at Thomayer Teaching Hospital’s Department of Respiratory Medicine in Prague, Czech Republic. The study was approved by the Ethics Committee of The Institute for Clinical and Experimental Medicine and Faculty Thomayer Hospital, June 27, 2012, ref. no. 1231/11(G11 06–26) and was based on a project entitled; “Biodegradable stents in the management of stenoses of large airways” (project NT 14146-3/2013); the project was awarded a special purpose grant with support from the Internal Grant Agency of the Czech Republic Ministry of Health.

### Inclusion criteria

Indications for implantation were functionally significant nonmalignant stenoses of the trachea where primary surgical resection was not possible. Every patient was reviewed by at least two interventional pulmonologists and a thoracic surgeon to determine the best therapeutic option. Bronchoscopy and computed tomography of the trachea were considered essential to confirm the diagnoses. All patients signed an informed consent form prior to undergoing the procedure. Contraindications included the presence of aero-digestive communication and pregnancy.

### Stents and implantation technique

**Fig. 1:**
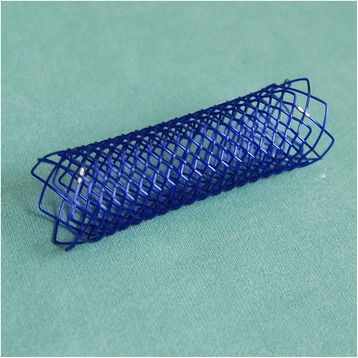
A polydioxanone tracheal stent with two golden radiopaque markers

The procedure utilized self-expandable, biodegradable, polydioxanone tracheal stents manufactured by ELLA-CS Ltd. (Hradec Králové, Czech Republic). The stents had the following dimensions (mm) (diameter x length): 18 × 50, 18 × 70, 20 × 50, and 20 × 70. The stents were knitted from polydioxanone monofilament, had sparse mesh near their ends and two gold radiopaque markers (Fig. 1). They were delivered in an airtight container, which was opened just prior to the procedure. The stents were manually loaded into the application apparatus, which consisted of a hollow plastic tube (sheath) and a flexible plastic piston inside the sheath. This apparatus worked as a pull-back delivery system, which was introduced through a rigid bronchoscope. The trachea was intubated with a rigid bronchoscope that was 43 cm in length and had an internal diameter of 8.5–11.0 mm (Karl Storz, Germany). Patients were placed under total intravenous anesthesia and jet ventilation. A flexible bronchoscope was used to measure the distance from the desired distal end location of the stent to the outer tube orifice. This distance was then marked on the delivery apparatus, which was introduced through a rigid tube to the desired depth of the distal end. After insertion, the position of the stent was determined and, if necessary, repositioned using rigid forceps. The final step was in-stent balloon dilation. In three patients, a thoracic surgeon secured the stent in place via external (percutaneous) fixation. In other words, one suture was passed through the stent, the tracheal wall, soft tissues, and skin; the suture was then knotted on the skin of the neck [19]. The suture was removed 2 to 3 weeks after implantation.

### Postoperative management and follow up

After insertion, patients were admitted to either the intensive care unit, or a standard ward, for at least 1 day of hospitalization. Inhalation of nebulized salbutamol or ipratropium was administered and intravenous methylprednisolone (40–80 mg) was usually administered on the day of the procedure. Alpha-escin (40 mg, p.o. tid) was recommended for at least 2 weeks to alleviate concomitant edema. The first bronchoscopy follow up was carried out during the first post-implantation week using topical anesthesia. Additional follow–ups (including bronchoscopy) were performed on a monthly or as-needed basis.

## Results

Between July 2013 and August 2014, six biodegradable stents were implanted in four patients with nonmalignant tracheal stenosis (Table 1). No technical difficulties occurred during the procedures, and all patients reported immediate relief from dyspnea. By December 2014, all stents had completely degraded. No serious or life-threatening events were recorded.

**Table 1:** Patients

Pt.	Etiology of narrowing	Localization in trachea	Type	Reduction of lumen cross-section (%)	Stents (ø × length, in mm)	External fixation of stents	Onset of rapid degradation (days)
1	Post-tracheotomy	upper and middle third	scar stricture and local malacia	50	18 × 70	Yes	88
2	Post-intubation	upper and middle third	scar stricture	75	18 × 70	No	90
3	Tracheomalacia associated with a. lusoria	middle and lower third	malacia	90	20 × 70	Yes	92
					20 × 70	Yes	85
4	Post–tracheotomy	upper third	hourglass	75	20 × 50	Yes	94
					20 × 50	Yes	86

### Patient 1

**Fig. 2:**
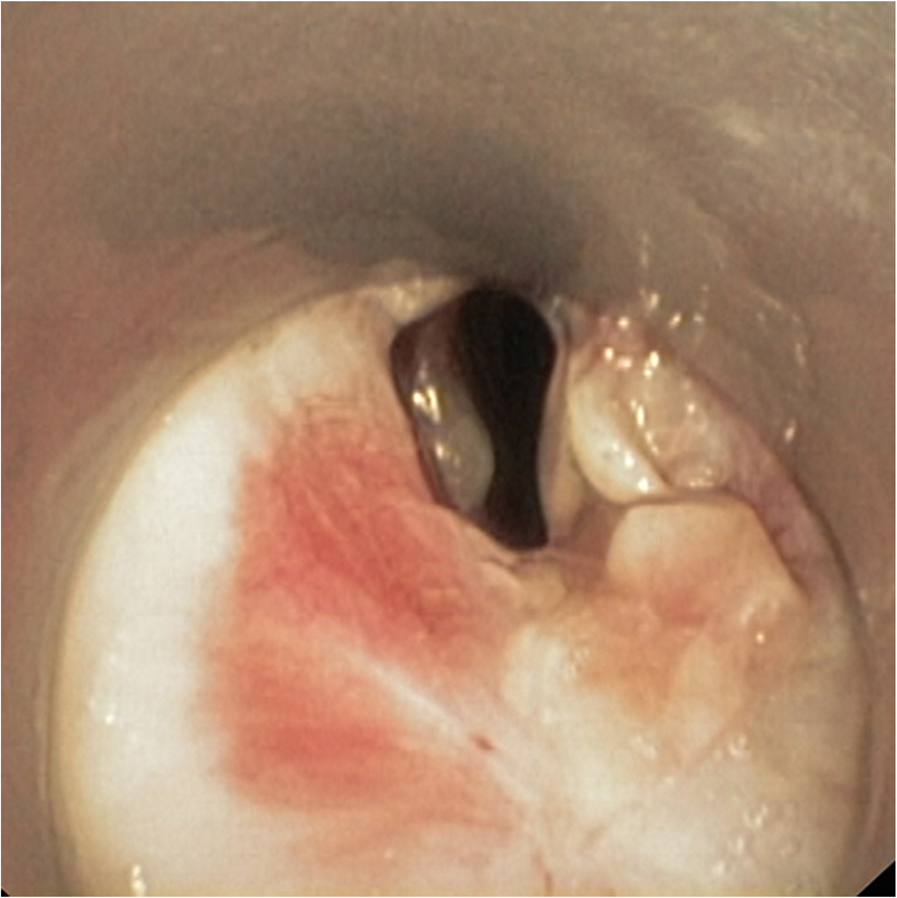
Severe post-tracheotomy stenosis before treatment

**Fig. 3:**
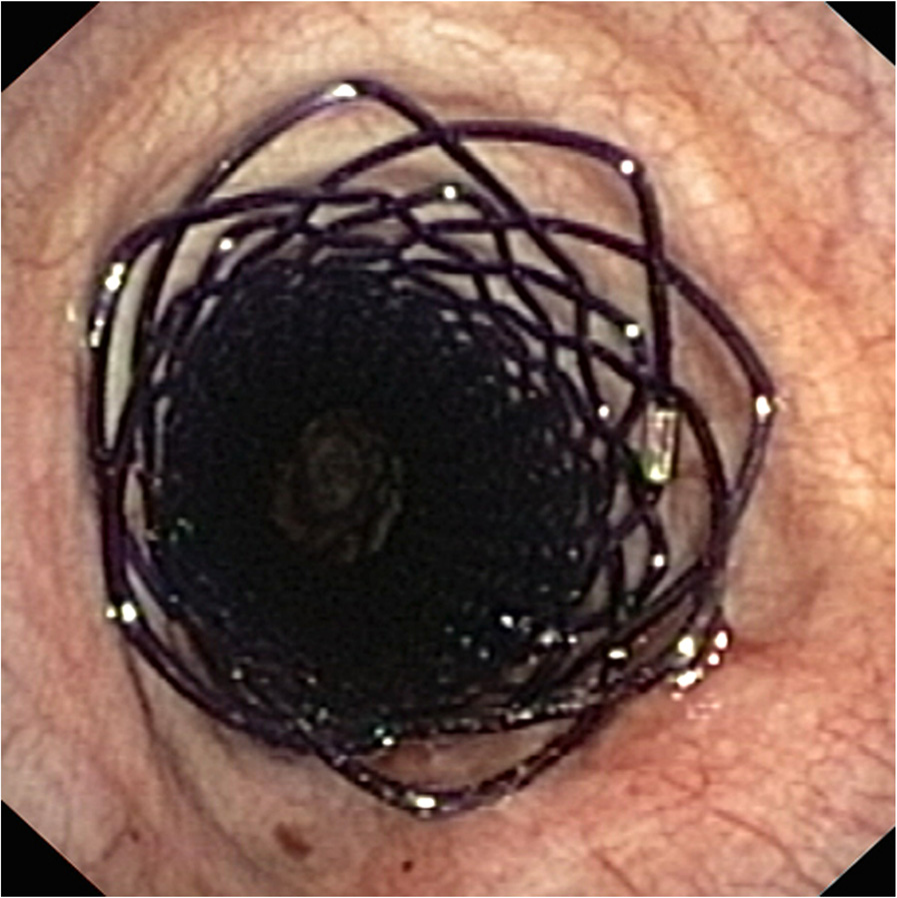
BD stent in trachea immediately after implantation

**Fig. 4:**
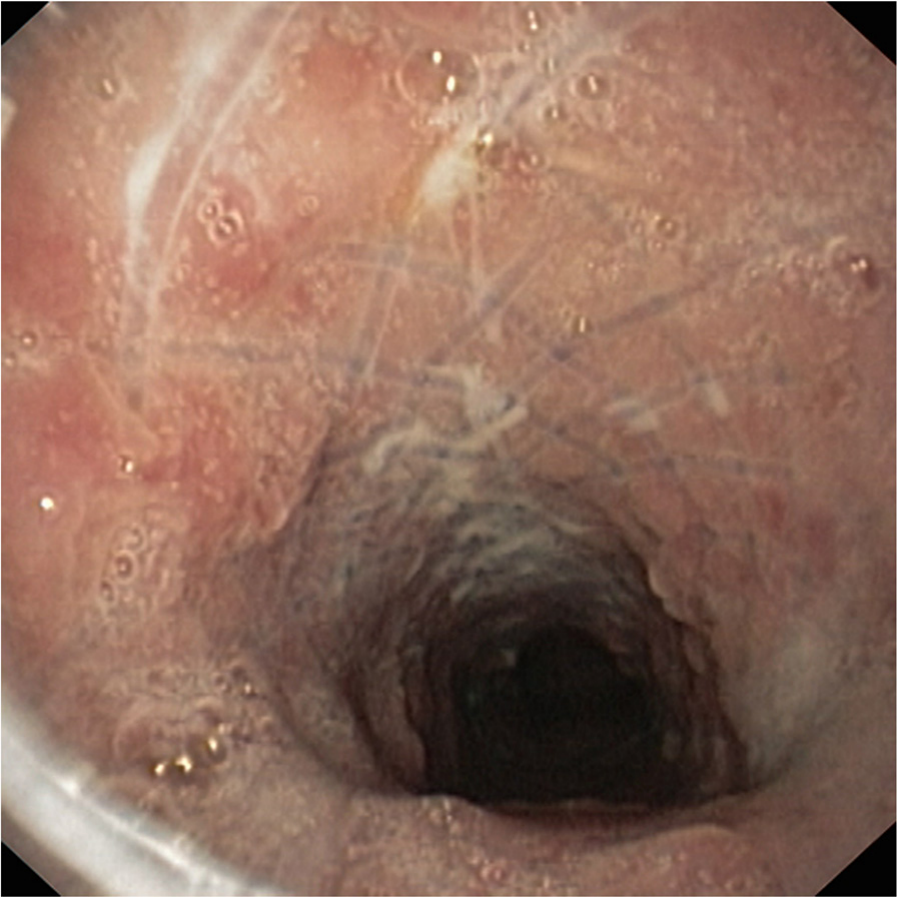
Proximal portion of the stent 3 months after implantation. Inflammation and hyperplasia of mucosa is seen, fibers are partially covered by the mucosa

**Fig. 5:**
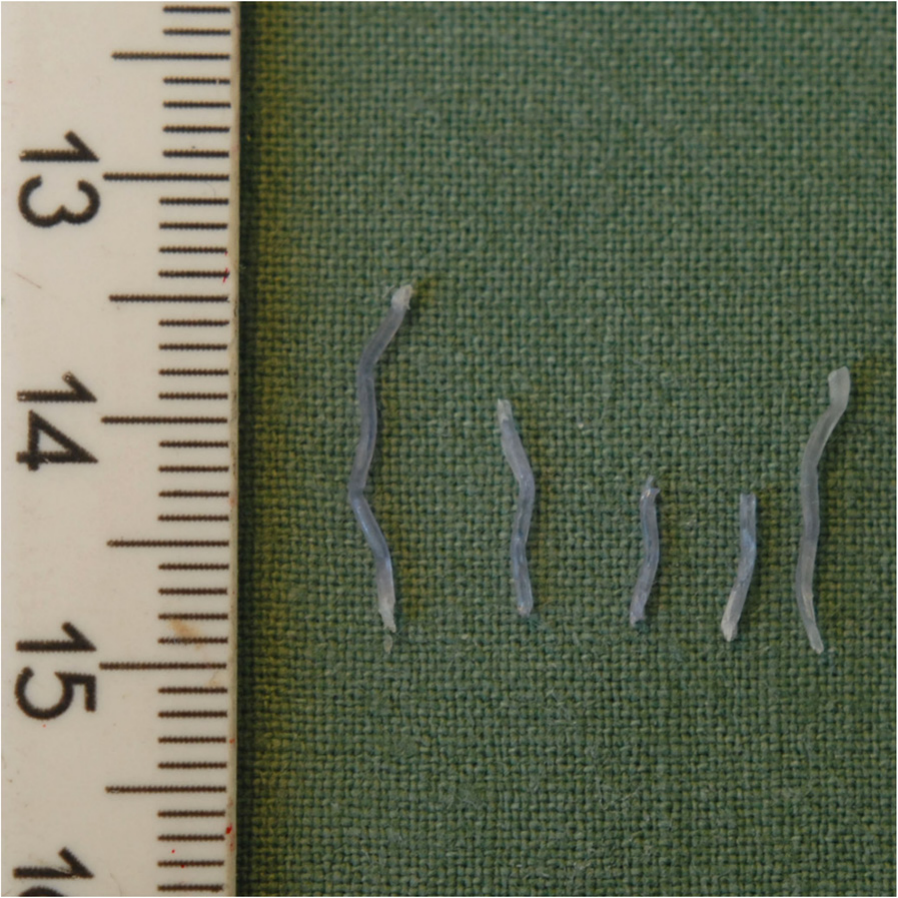
Bits of fibers coughed up by one of the patients

**Fig. 6:**
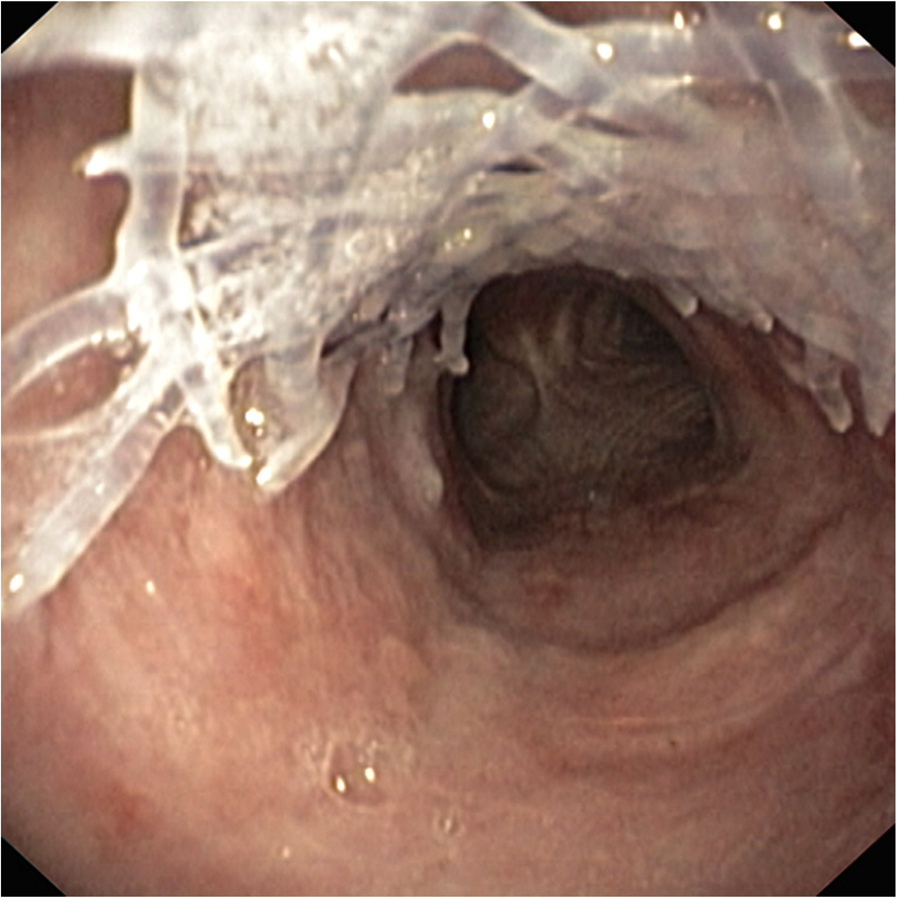
Three months post-implantation, distal part of the stent is missing

**Fig. 7:**
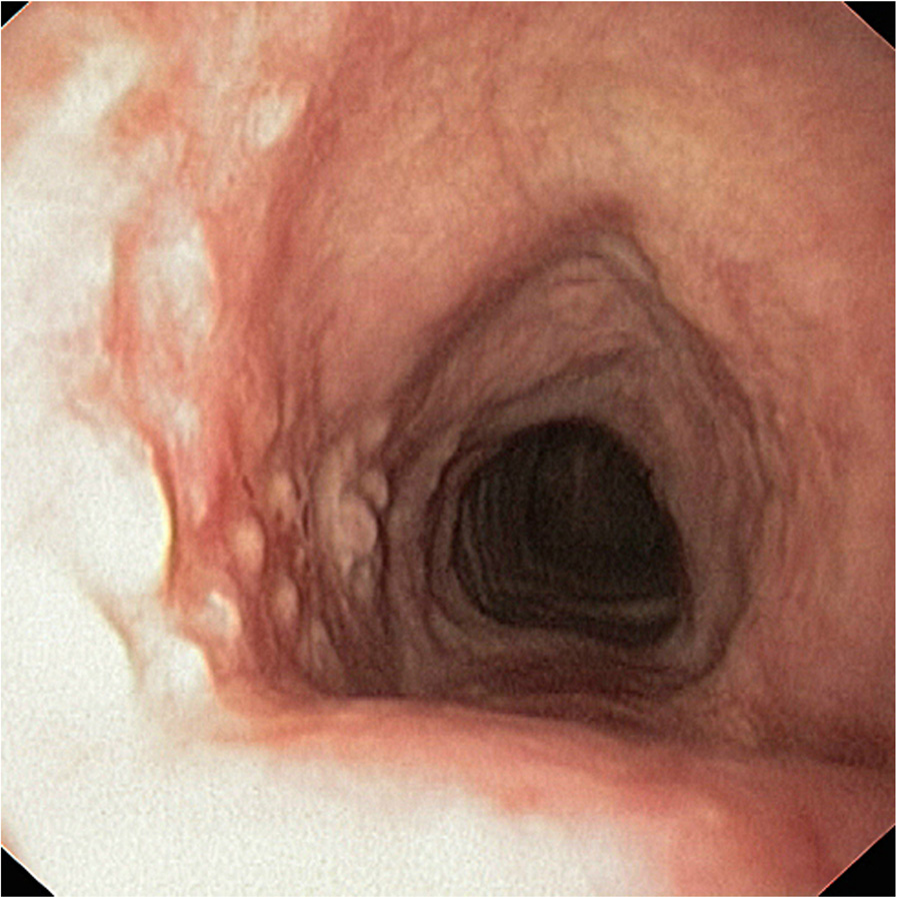
Seven months after implantation; cobblestone pattern of mucosal hyperplasia represents imprint of degraded stent

A 59-year-old man had post-tracheostomy stenosis. He had undergone a heart transplantation, which had been followed by numerous complications requiring prolonged mechanical ventilation. An irregular stenosis with two fractured tracheal cartilages was found; this resulted in an 80 % lumen reduction and a stricture length of 5 cm (Fig. 2). The patient was unable to undergo further surgery without significant risk. In March 2013, balloon dilation was performed and a straight silicone stent was inserted. The patient suffered from frequent exacerbations involving dyspnea caused by mucus impaction and a persistent cough despite appropriate supportive treatment. In September 2013, the Dumon stent was replaced with a BD stent. After removal of the silicone stent, a significant residual stenosis (lumen reduction of 50 %) was still present. The insertion of the BD stent went smoothly and tracheal patency was excellent (Fig. 3). However, the stent appeared to be easily movable and given our lack of previous experience, a thoracic surgeon was enlisted to fix the stent externally to prevent dislocation. The patient reported marked improvement of his dyspnea and fewer difficulties with expectoration; no further occurrences of intermittent worsening of dyspnea due to mucus stagnation were observed. Follow–up bronchoscopy revealed minor irritation of the tracheal mucosa, hypertrophy between the stent fibers, and a small degree of non-purulent secretion (Fig. 4). Three months after implantation, the patient began to cough up small bits of fibers (no longer than 15 mm) (Fig. 5). This episode lasted approximately 10 days, and the patient described the process as “very unpleasant”; however, his dyspnea did not worsened. Bronchoscopy found that the lower part of the stent, which previously had poor contact with the mucosa due to a spacious trachea, had disappeared (Fig. 6). The remainder of the stent, with its fibers nearly encased in hyperplastic mucosa, had not been breached. Neither restenosis, nor any other situation necessitating treatment of any kind, occurred. Five months after implantation, the remainder of the stent was still observed in situ. We noted a significant reduction in inflammation and fewer secretions. Seven months after implantation, no biodegradable materials were found in patient’s trachea; however, the cobblestone pattern that the stent mesh had left on the mucosa was still apparent (Fig. 7). Some degree of small, insignificant narrowing remained, but further intervention was not needed.

### Patient 2

A 52-year-old woman suffered from a complex post-intubation stenosis. She was first referred to our department in 2012 and a silicone stent was inserted to treat a 4 cm long irregular narrowing that affected the upper and middle portions of her trachea. Unabated alcohol intake, together with advanced hepatic cirrhosis, contraindicated tracheal resection. With the exception of minor expectoration difficulties, she did well with the silicone stent until November 2013, when she presented with a growth of granulation tissue and mucosal swelling above the proximal end of the stent. After removal of the stent, a significant residual stenosis persisted despite laser resection of the granulation and balloon dilation. We then inserted a BD stent, which very nicely reopened the narrowed segment; the patient reported a reduction in coughing and expectoration compared to the silicone stent. Thirteen weeks after implantation, the patient presented with dyspnea and stridor, together with a history consistent with a respiratory infection. We found significant narrowing caused by granulations protruding between the holes of the stent mesh. The stent integrity had been breached and several fibers were projecting into the lumen. All visible stent components were removed, followed by partial laser resection of the granulation and balloon dilation. Ultimately, the patient was referred for tracheostomy due to residual narrowing that was primarily caused by edema. One week later, the inflammation receded and the patient was successfully decannulated. Although some insignificant narrowing remained, no further interventions were needed.

### Patient 3

A 70-year-old man had severe tracheomalacia associated with arteria lusoria. Despite having undergone surgical treatment for the anomaly (reimplantation of the aberrant artery into the right common carotid artery) at a different hospital in 2012, a nearly-complete collapse of the lower and middle trachea persisted. As such, a tracheostomy appeared to be the patient’s only option. He complained of a persistent cough, stagnated secretions, and recurrent aspiration of small amounts of fluid when swallowing. A significant narrowing distal to the end of the tracheostomy tube was observed. However, the patient was not able to tolerate a longer tube, which proved too difficult for him to keep clean. In May 2014, a BD stent was implanted distal to the level of the tracheostomy. Since the stent reached just below the level of the tracheostomy it was secured using external fixation, and the tracheostomy tube was left in situ. The patient reported significant relief and was able to expectorate. Some degree of mucosal hypertrophy and minor secretion stagnation were observed. Thirteen weeks after implantation, the stent was found to be partially absorbed and recurrent narrowing was observed. Another BD stent with the same dimensions was introduced. It, too, induced some degree of mucosal hypertrophy and granulation growth, but the overall effect that persisted after the second stent’s absorption was better than had been observed prior to BD stenting: a more spacious lumen that was less prone to collapse had been achieved.

### Patient 4

A 59-year-old man who had previously survived a car accident presented with a severe, hour-glass shaped, post-tracheostomy stenosis in the upper trachea. Surgical resection was considered too risky due to the patient having coronary artery disease, as well as his recent recovery from polytrauma. After balloon dilation and insertion of a BD stent, the patient experienced immediate and excellent clinical benefits. At the 1-month post-implantation follow-up, several islands of granulation tissue were observed to be growing through the stent mesh. These led to some reduction of the tracheal lumen, but not more than 30 %. The patient was prescribed inhaled corticosteroids (budesonide dry powder inhaler, 400 mcg, b.i.d.) and clarithromycin (p.o. for 3 weeks). At the next follow up, the reduction had receded slightly and the patient remained well. On the 88th post-implantation day, he started to cough up pieces of stent fiber and complained of dyspnea. An examination found re-stenosis due to granulations and recurrent scaring, which necessitated balloon dilation and insertion of a new BD stent. Four months later, the patient was in good health and no significant tracheal narrowing was observed during absorption of the second stent.

## Discussion

We inserted six BD stents into four patients with tracheal narrowing. This treatment of adult patients was novel, and there have only been a few cases in which polydioxanone stents were used to maintain airway patency: Lischke et al. published results from six patients with bronchial stenosis after lung transplantation [15]; Vondrys et al. described treatment in four children with tracheal narrowing [16]; and BD stents have recently been used in children [17, 18].

Stents from biodegradable materials only provide temporary support. Their use is especially indicated in cases where time-limited support for healing is needed. Nonmalignant stenoses may stabilize over time as the tissue stiffens. The absence of a recurrent post-intubation stenosis after silicone stent removal at 1 year ranges from 40 % (when the stent was in place for 6 months) to more than 60 % (when it remained in place for 18 months) [3]. It is worth noting that both of the above-mentioned time periods exceed the degradation time of polydioxanone airway stents; however, this time can be extended through multiple stentings, which has been shown to be effective [15, 16]. The airway remodeling can be achieved using BD stents; moreover, we believe the induction of mucosal hyperplasia that often accompanies BD stenting can contribute to stabilization of the narrowing and the total time needed for mechanical support in the airways, including multiple implantations of BD stents, may be shorter.

The implantation of all BD stents went smoothly and technical success was achieved in each case. We utilized a rigid bronchoscope and operated under visual control. While this method is suitable in adults with large, spacious airways, fluoroscopic guidance is recommended in the smaller airways of children [16]. In three patients we used external fixation of the stent due to concerns over dislocation [19]. This procedure was simple and did not represent any additional risk to the patient. Fixation of BD stents is recommended when we suspected that the stent was likely to slip out of position, e.g. when there is a tough, irregular funnel-shaped stenosis or stenosis in the upper portion of trachea. Of course fixation only took place when it was technically possible, i.e. at least part of the stent was located in the extrathoracic trachea. Longer BD stents can be used to address this particular issue in distal tracheal stenosis. As mentioned above, fixation is temporary, and the suture is removed 2 to 3 weeks after implantation, which is long before the start of the degradation process and before radial strength is compromised. Fixation simply holds the stent in place until some of the stent fibers are encased in hyperplastic mucosa.

All patients reported relief from their symptoms. During the post-implantation period, patients only complained of coughing; however, those who had previously been treated with silicone stents reported coughs of reduced intensity and decreased expectoration. Mild irritation of the tracheal mucosa, mucosal hyperplasia, and stagnation of non-purulent secretions were also observed in our patients. Novotny et al. [10] described histopathologic changes in tracheal mucosa after polydioxanone-stenting in rabbits. The most intensive period of inflammation was observed 5 weeks after implantation, with the majority of inflammation occurring during the first 3 months after implantation. Rapid deterioration of stent integrity began approximately 12–13 weeks (90 days) after implantation. This was followed by an apparent reduction in inflammatory changes; while some remnants of the stent were still observed in situ 5 months after implantation. Findings of a similar nature have also been reported by other authors [15, 16].

We used BD stents on only four patients, two of whom were naive to previous stenting. Issues that appeared in patients with previously-inserted silicone stents encouraged us to replace them. In both cases, significant narrowing persisted after removal of silicone stents; therefore, we do not think that the previous stenting procedures substantially influenced our results.

Expectoration of stent particles is a concomitant and predictable situation [15] that indicates the start of rapid degradation. Given their relatively small dimensions, we believe that the remnants of BD stents are unlikely to occlude the trachea (Fig. 5); nonetheless, this symptom should lead clinicians to perform a follow-up bronchoscopy if restenosis (due to the character of the tracheal disease), is expected. Nevertheless, these cases can be prevented with early re-stenting. The onset of rapid degradation was relatively constant (Table 1), which corresponds to previous observations [10, 15, 16]. Serio and colleagues [18] published unsatisfactory experiences with polydioxanone stents that were especially associated with a lack of radial force; however, they were working with stents having a completely different structure. We recorded growth of exophytic granulations, which led to restenosis in two patients, however we suspect that respiratory infections to have been the trigger of these occurrences. The growth of granulations was a bit disappointing since we had expected perfect biocompatibility with polydioxanone stents and minimal mucosal inflammatory reactions. Polydioxanone fibers can induce granulations as described by Lischke et al. and Vondrys et al. [15, 16], but other risk factors, such as infections and gastroesophageal reflux, can also contribute to their development. These conditions are sometimes difficult to identify and when revealed, they deserve thorough treatment. Nevertheless, clinicians need to be ready to deal with granulations during treatment with BD stents. In our particular cases, they were resolved via endoscopic removal, balloon dilation, and re-stenting.

On the other hand, fibrosing processes and chondrification are cornerstones for the stabilization of a stenosis. The presence of a biodegradable stent can positively influence the buildup of cartilage [11]. We assume mucosa hyperplasia induced by BD stents to have been an independent factor that contributed to the augmentation of the malatic trachea.

## Conclusions

Biodegradable tracheal stents are novel types of tracheal support devices that appear capable of treating benign tracheal stenosis; however, their effectiveness has yet to be verified. Having limited experience based on an analysis of four patients, we must emphasize some important challenges. Due to time-limited mechanical support of the trachea, re-stenosis may develop during degradation of the stent. Therefore, success depends on follow-ups by an interventional pulmonologist and good patient compliance. BD stents are not free of side effects: i.e., they may induce growth of granulations and expectoration of stent particles, which is a specific BD stent related phenomenon; which, as would be expected, is perceived negatively by patients. While not dangerous, it does indicate the onset of rapid degradation of the stent and associated effects. Repeated bronchoscopy and re-stenting may be necessary if tracheal narrowing remains.

Despite these disadvantages, stenosis remodeling can be achieved leaving the patient without any artificial supportive devices in their airways. The induction of inflammation followed by the development of mucosal hypertrophy may lead to difficulties, but may also result in exploitable stabilizing effects, particularly in patients with malacia. Based upon our limited experience, we are unable to postulate an unequivocal statement about this novel method, but we believe that these types of stents may overcome the disadvantages of silicone and metal stents. Further research in this field is needed to confirm our hypothesis. Future modifications in stent design and materials might further improve BD stent characteristics, their biological compatibility, and effects.

## Ethics approval and consent to participate

The study was approved by the Ethics Committee of The Institute for Clinical and Experimental Medicine and Faculty Thomayer Hospital, June 27^th^, 2012, ref. no. 1231/11(G11 06-26). All patients signed an informed consent prior to participation in the study.

## Consent for publication

All patients signed an agreement that allowed publication of their clinical data. The agreement was part of informed consent.

## Availability of data and materials

The datasets supporting the conclusions of this article are available in The Research and Development and Innovation Information System of the Czech Republic [R&D IS of the Czech Republic, http://www.isvav.cz/projectDetail.do?rowId=NT14146]. This database now contains a limited amount of data, however it will be fully completed by October 2016 after the research project has concluded. Other supporting datasets are available in AEPress database [http://www.elis.sk/download_file.php?product_id=4251&session_id=5b85cf03c209700a4304658df30cf3a5 ].

## Abbreviations

b.i.d.: Bis in die
BD: Biodegradable
cm: Centimeter
Fig.: Figure
Ltd.: Limited company
mcg: Microgram
mg: Milligram
mm: Millimeter
p.o.: Per os
pH: Potential of Hydrogen
t.i.d.: Ter in die
